# The Retinoblastoma Tumor Suppressor Transcriptionally Represses Pak1 in Osteoblasts

**DOI:** 10.1371/journal.pone.0142406

**Published:** 2015-11-10

**Authors:** Bernadette Sosa-García, Viviana Vázquez-Rivera, Jonathan N. González-Flores, Brienne E. Engel, W. Douglas Cress, Pedro G. Santiago-Cardona

**Affiliations:** 1 Department of Basic Sciences, Biochemistry Division, Ponce Health Science University, Ponce, Puerto Rico; 2 Molecular Oncology and Thoracic Oncology Departments, H. Lee Moffitt Cancer Center and Research Institute, Tampa, FL, United States of America; NCMLS, Radboud University Nijmegen Medical Center, NETHERLANDS

## Abstract

We previously characterized the retinoblastoma tumor suppressor protein (Rb) as a regulator of adherens junction assembly and cell-to-cell adhesion in osteoblasts. This is a novel function since Rb is predominantly known as a cell cycle repressor. Herein, we characterized the molecular mechanisms by which Rb performs this function, hypothesizing that Rb controls the activity of known regulators of adherens junction assembly. We found that Rb represses the expression of the p21-activated protein kinase (Pak1), an effector of the small Rho GTPase Rac1. Rac1 is a well-known regulator of adherens junction assembly whose increased activity in cancer is linked to perturbations of intercellular adhesion. Using nuclear run-on and luciferase reporter transcription assays, we found that Pak1 repression by Rb is transcriptional, without affecting Pak1 mRNA and protein stability. Pak1 promoter bioinformatics showed multiple E2F1 binding sites within 155 base pairs of the transcriptional start site, and a Pak1-promoter region containing these E2F sites is susceptible to transcriptional inhibition by Rb. Chromatin immunoprecipitations showed that an Rb-E2F complex binds to the region of the Pak1 promoter containing the E2F1 binding sites, suggesting that Pak1 is an E2F target and that the repressive effect of Rb on Pak1 involves blocking the trans-activating capacity of E2F. A bioinformatics analysis showed elevated Pak1 expression in several solid tumors relative to adjacent normal tissue, with both Pak1 and E2F increased relative to normal tissue in breast cancer, supporting a cancer etiology for Pak1 up-regulation. Therefore, we propose that by repressing Pak1 expression, Rb prevents Rac1 hyperactivity usually associated with cancer and related to cytoskeletal derangements that disrupt cell adhesion, consequently enhancing cancer cell migratory capacity. This de-regulation of cell adhesion due to Rb loss could be part of the molecular events associated with cancer progression and metastasis.

## Introduction

Inactivation of the retinoblastoma tumor suppressor protein (Rb) occurs with high frequency as one of the early events in human tumorigenesis [1–5]. Pervasive Rb inactivation in an oncogenic context is natural given that Rb has been characterized predominantly as a cell cycle repressor, specifically as the main regulator of the G_1_-S transition checkpoint [1, 2]. Normal cells respond to anti-proliferative signals by de-phosphorylating Rb with consequent activation of Rb function at the G_1_ phase [1, 2, 6, 7]. Active Rb then binds E2F transcription factors and abrogates their ability to induce S-phase-related gene expression [1, 2]. This diverts cells to G_0_ rather than allowing their progression to the S phase. In contrast, Rb inactivation de-regulates the cell cycle and renders cells incapable of exiting a proliferative state.

The fact that the pathway centered on Rb is impaired in most human cancers [1, 4, 8] suggests that this pathway is a prominent anti-oncogenic barrier that cells must overcome in the transformation process. Rb can be indirectly inactivated by chronic hyperphosphorylation in tumors that bear mutations targeting genes coding for other Rb pathway components such as CDK4, cyclin D, and p16ink4a [8]. There are human tumor types such as retinoblastomas, osteosarcomas, and small-cell lung carcinomas in which the *RB1* gene coding for Rb is mutated, usually resulting in complete abrogation of Rb expression [4]. Research on these Rb-deficient tumors revealed an additional Rb function in cell adhesion, namely, the induction of the assembly at the cell membrane of catenin- and cadherin-containing adherens junctions involved in cell-to-cell contacts. Rb-deficient tumors show diminished expression of cadherins and catenins, which also fail to stably anchor to the cell membrane and show a rather diffuse cytoplasmic distribution [9, 10, 11]. These studies linked Rb for the first time with a non-traditional, non-cell cycle-related role by implicating it in the assembly of adherens junctions. Based on these early studies, promoting cell-to-cell contacts could be an additional Rb-induced tumor suppressive mechanism, which when engendered together with the capacity to block the cell cycle, could enhance the tumor suppressive power of Rb.

We are currently deepening the mechanistic understanding of the relation of Rb to cell adhesion using osteoblasts, which require both functional Rb and cell-to-cell contact-mediated spatial cues in order to differentiate and produce bone [5, 12, 13, 14, 15]. We previously reported that conditional Rb deletion in osteoblasts by Cre-mediated excision of the *RB1* gene results in aberrant expression of a variety of cell adhesion genes, including integrins and cadherins, involved in the disruption of adherens junctions, and in the disruption of cell-to-cell and cell-to-substrate adhesion [16, 17]. We reported that the capacity of Rb to promote adherens junction assembly is mediated in part by its capacity to promote the activity of merlin (Moesin-Ezrin-Radixin-Like Protein) tumor suppressor [16]. Merlin is a membrane-bound cytoskeleton adapter protein that stabilizes adherens junctions by anchoring them to the cortical actin cytoskeleton [18, 19]. We showed that, in the absence of Rb, p21-activated protein kinase (Pak1), which is an effector of the small Rho GTPase Rac1, becomes upregulated with consequent phosphorylation of merlin serine 518 by Pak1, leading to merlin detachment from the cell membrane and destruction of adherens junctions [16]. Therefore, Rb seems to promote adherens junction assembly at the cell membrane by blocking the inactivating phosphorylation of merlin by Pak1.

This article specifically focuses on Rb repression of Pak1, a mechanism important for adherens junction assembly and stabilization. By repressing Pak1 expression, Rb could prevent the Rac1 hyperactivity that has been previously related to the disruption of adhesive structures in epithelial cells [20], a process that can exacerbate metastasis by promoting detachment of tumor cells from the primary site. This deregulation of cell adhesion due to Rb loss could be part of the molecular events associated with cancer progression and metastasis.

## Results

### Silencing of Pak1 expression in Rb-deficient cells partially restores adherens junction structures

**Fig 1:**
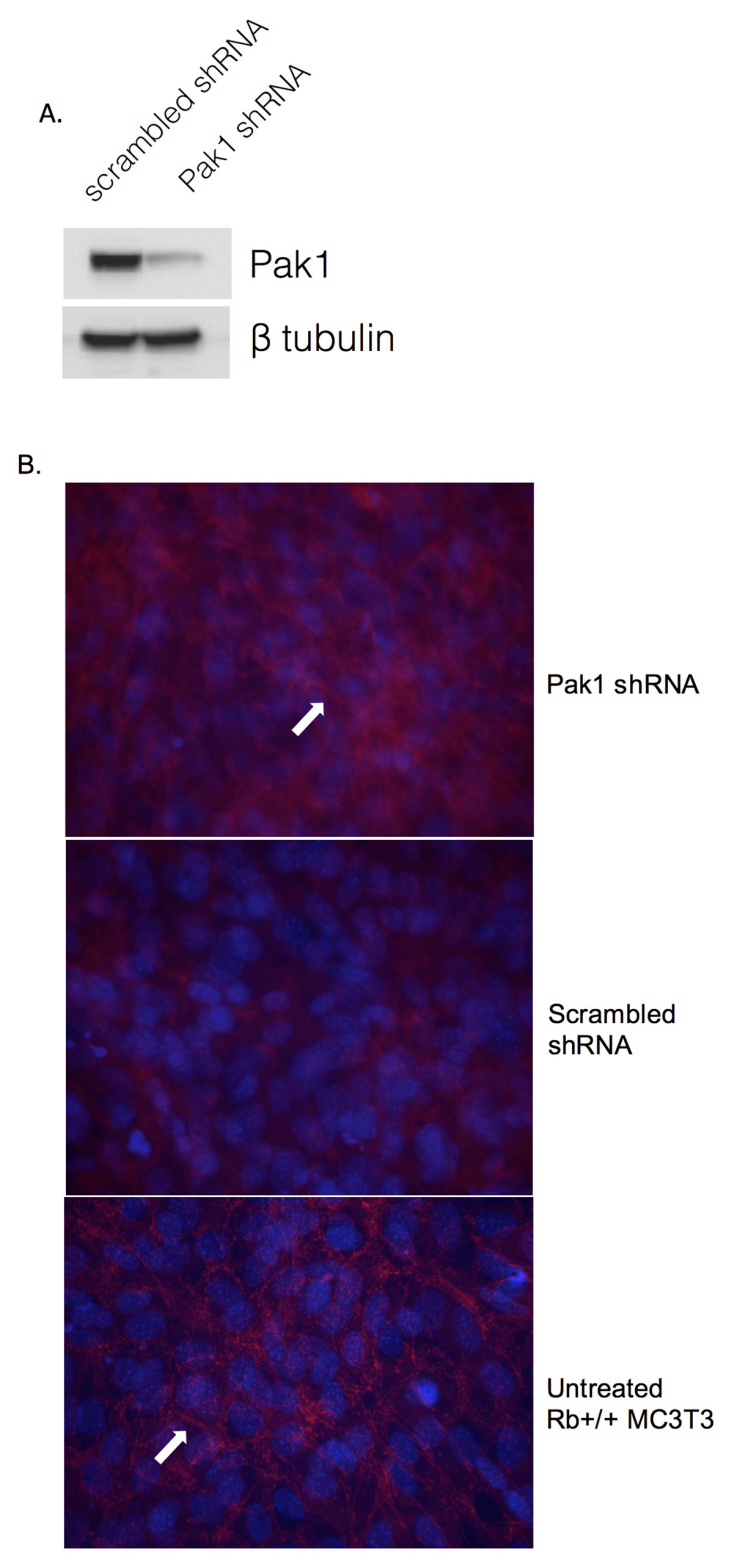
Pak1 silencing partially restores adherens junctions. **(A)** Immunoblot analysis showing reduced Pak1 expression in Rb-/- MC3T3 cells infected with an adenovirus vector carrying an RNAi against Pak1, relative to a scrambled vector control. **(B)** Immunofluorescence labeling showing re-establishment of beta-catenin presence in the intercellular spaces after infection of Rb-/- MC3T3 cells with adeno-Pak1 RNAi (*top panel*, *arrow*), relative to Rb-/- MC3T3 cells infected with a scrambled control-carrying adenovirus (*middle panel*). Untreated Rb+/+ MC3T3 cells showing intercellular beta-catenin labeling are shown for comparison (*bottom panel*, *arrow*).

We previously showed that Rb-expressing MC3T3 osteoblasts have significantly diminished Pak1 mRNA and protein steady-state levels relative to their Rb-deficient counterparts [16], suggesting that Rb exerts a repressive action on Pak1 expression. In order to further confirm that Pak1 expression negatively affects the formation of adherens junctions, an adenoviral vector-based Pak1 RNAi approach was used to decrease Pak1 levels in Rb-deficient osteoblasts lacking adherens junctions. Infection of Rb-/- MC3T3 cells with Pak1 Adeno-RNAi dramatically decreased Pak1 expression relative to an Adeno-scrambled control, as assessed by Pak1 immunoblot (Fig 1A). Next, we performed immunofluorescence labeling using antibodies against Beta-catenin to detect the presence of adherens junctions as indicated by the presence of beta-catenin in intercellular spaces. As shown in Fig 1B, Pak1 RNAi infection of Rb-/- MC3T3 cells increased the intensity of beta-catenin labeling in intercellular spaces, suggesting a partial restoration of adherens junctions (*top panel*, *arrow*), compared to Rb-/- MC3T3 cells infected with scrambled control adenovirus (*middle panel*). Beta-catenin staining in Pak1 RNAi infected Rb-/- MC3T3 cells shows a similar pattern to the Beta-catenin staining observed in untransfected Rb+/+ MC3T3 cells (*bottom panel*, *arrow*).

### Rb Represses Transcription of the *Pak1* Gene

**Fig 2:**
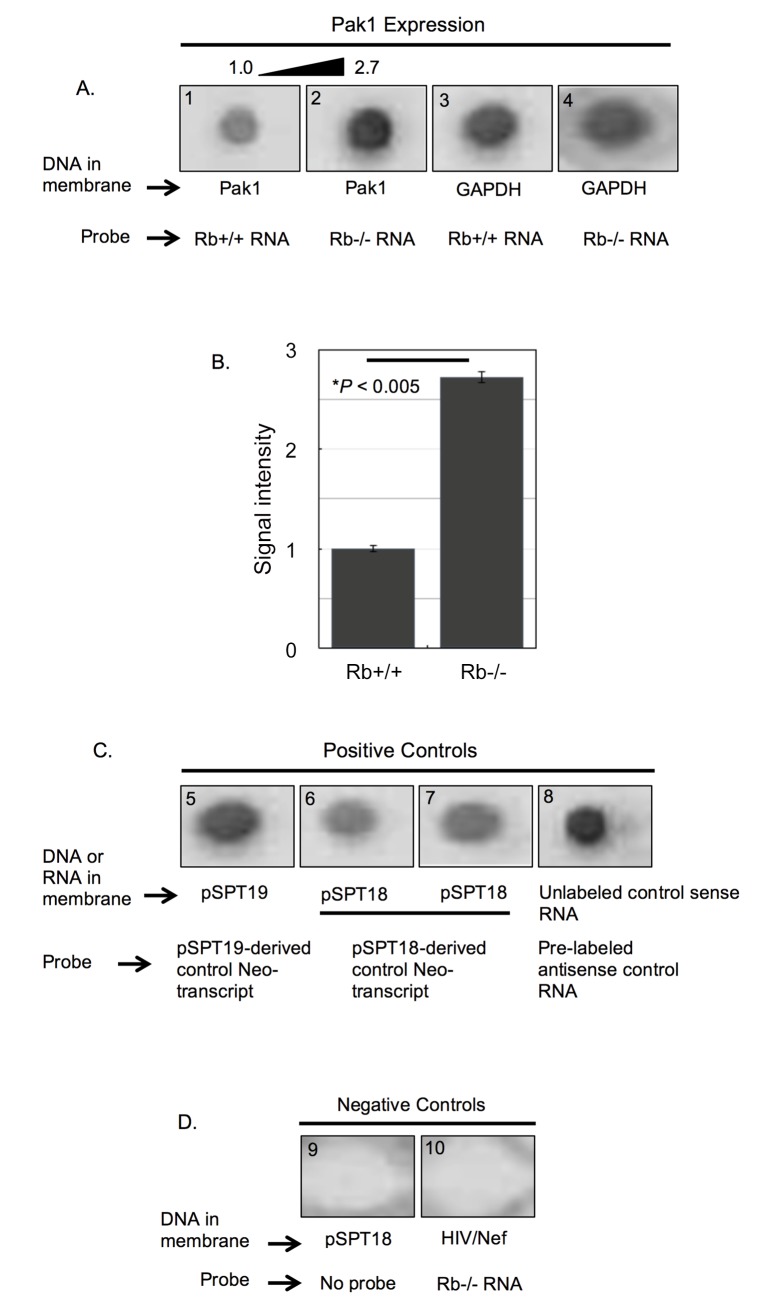
Rb represses the transcription rate of the Pak1 gene. **(A)** Representative nuclear run-on assay from a triplicate experiment showing hybridization of Rb+/+ (*blot 1*) and Rb-/- (*blot 2*) DIG-labeled mRNA probes to a Pak1 cDNA immobilized in a membrane. These two probes were also hybridized to GAPDH cDNAs (*blots 3* and *4*). Hybridization signal intensity was stronger with the Rb-/- probe, indicating higher transcriptional rate of the Pak1 gene in Rb-/- nuclei. **(B)** To determine the differences in signal intensity, the chemioluminiscence in the membranes was quantified and analyzed using BioRad’s Quantity 1 Imaging Software and normalized against the signal intensities obtained when hybridizing to the GAPDH cDNAs (blots 3 and 4 in **A**). This quantitative analysis showed a statistically significant 2.7-fold increase in the signal intensity of the Rb-/- hybridization over the Rb+/+ hybridization. Each bar represents the mean of at least 3 independent experiments (±SE of the mean), with P < 0.005. **(C)** Strong signal intensities were observed in all positive controls in which pSPT19- and pSPT18-derived DIG-labeled control transcripts were hybridized to the parental plasmid from which they were transcribed (*blots 5–7*), as well as a pre-labeled antisense control RNA hybridized to its corresponding unlabeled sense RNA (*blot 8*). **(D)** As negative controls, either the probe was omitted from the hybridization reaction (*blot 9*) or a DIG-labeled mRNA probe from osteoblasts was hybridized to an irrelevant plasmid encoding the lentiviral protein Nef (*blot 10*).

Given the well-studied function of Rb as a transcriptional regulator, we asked whether Pak1 repression by Rb occurs at the transcriptional level. To test this, nuclear run-on assays compared Rb+/+ and Rb-/- MC3T3 osteoblasts in terms of the transcriptional rate of the *Pak1* gene. Briefly, Rb+/+ and Rb-/- MC3T3 osteoblasts were cultured until confluence after which nuclei were harvested to halt transcription due to nucleotide depletion. Transcription by purified nuclei was then resumed *in vitro* in the presence of a nucleotide mix that included digoxigenin (DIG)-11-UTP as a label. DIG-labeled mRNA probes from Rb+/+ and Rb-/- osteoblasts were then hybridized to linearized and gel-purified Pak1 cDNA immobilized on a nylon membrane. Nuclear run-ons in Fig 2A showed decreased signal intensity when the Pak1 cDNA was hybridized to the Rb+/+ probe (*blot 1*), relative to hybridization to the Rb-/- probe (*blot 2*). In addition, Rb+/+ and Rb-/- DIG-labeled mRNA probes were hybridized to cDNA coding for the housekeeping gene glyceraldehyde-3-phosphate dehydrogenase (GAPDH) (Fig 2A, *blots 3* and *4*) to rule out unequal cDNA loading of the dot blot apparatus or unequal cDNA binding to the membrane. Quantification of signal intensity showed that the Rb-/- signal was 2.7-fold stronger than the Rb+/+ signal (Fig 2B), after normalization of GAPDH signal intensity (*blots 3* and *4* in Fig 2A). This magnitude is remarkably similar to the one we previously reported for increased levels of Pak1 mRNA and protein in Rb-/- relative to Rb+/+ osteoblasts [16]. In nuclear run-ons, positive controls were pSPT19- and pSPT18-derived DIG-labeled control transcripts hybridized to the same parental plasmid from which they were transcribed (*blots 5–7* in Fig 2C), as well as a pre-labeled antisense control RNA hybridized to its corresponding unlabeled sense RNA (*blot 8* in Fig 2C). As negative controls, either a probe was omitted from the hybridization reaction (*blot 9* in Fig 2D) or a DIG-labeled mRNA probe from osteoblasts was hybridized to an irrelevant plasmid encoding the lentiviral protein Nef (*blot 10* in Fig 2D).

### Rb does not affect Pak1 mRNA or protein stability

**Fig 3:**
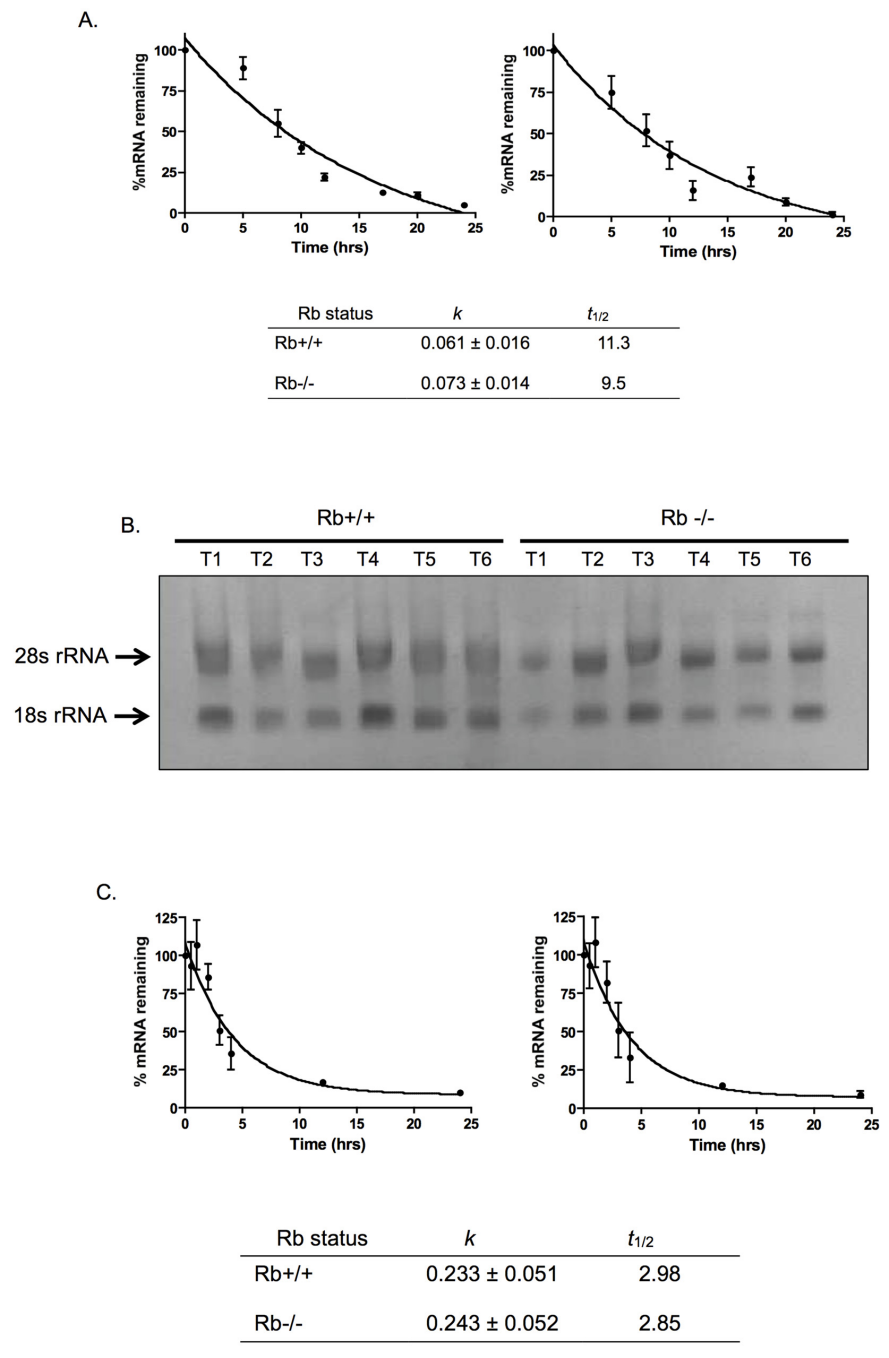
Rb does not affect Pak1 mRNA or protein stability. **(A)** Rb+/+ and Rb-/- MC3T3 cells were incubated with the transcriptional inhibitor actinomycin D, and Pak1 mRNA levels were assessed by qRT-PCR at 0, 5, 8, 10, 12, 17, 20, and 24 hours after actinomycin D addition. The graph shows percent of Pak1 mRNA, normalized against β-actin mRNA, remaining as a function of time incubated in actinomycin D relative to T_0_ (no actinomycin D), which was arbitrarily assigned a value of 100%. Data were analyzed by nonlinear regression, and the Pak1 mRNA *t*_1/2_ was calculated from the first-order decay constant (*k*) obtained with the PRISM software program (GraphPad). Rb+/+ and Rb-/- MC3T3 cells show similar decay constants (*k*) and *t*_1/2_ and comparable to the previously reported 10 hours [21], suggesting that these parameters are not affected by Rb status. **(B)** Samples of total RNA from Rb+/+ and Rb-/- cells were collected at various time intervals (T1 –T6) during the time course of the experiment and run in a 1.4% agarose gel containing formaldehyde. Ethidium bromide staining revealed a 2:1 ratio of 28S-to-18S rRNA and no visible signs of degradation, indicating high quality of the RNA without any signs of degradation and suggesting that the measured degradation of Pak1 mRNA is not a reflection of a general degradation of total RNA. **(C)** Kinetics of decay of the myeloid cell leukemia-1 (MCL1) mRNA used as a reference. The experiment was performed essentially as described in **(A)**. In both Rb+/+ and Rb-/- MC3T3 cells, MCL1 mRNA had a *t*_1/2_ of approximately 3 hours, which is what has been reported for this mRNA [22], and the *k* was unaffected by Rb status. **(D)** Immunoblot analysis of Pak1 protein levels at different time points after addition of 10 mg/mL puromycin (*top*) and graph showing changes of Pak1 protein expression levels as a function of time incubated in puromycin (*bottom*). The time points analyzed were 0, 2, 5, 8, and 11 hours. Drug toxicity precluded the analysis of time points beyond 11 hours. Pak1 levels were normalized against α-tubulin expression. No significant changes were observed in terms of Pak1 protein degradation when comparing Rb+/+ and Rb-/- MC3T3 cells, suggesting that Rb does not affect Pak1 protein stability, at least within the time points evaluated.

mRNA and protein stability represent two additional levels of regulation of gene expression that could impinge upon protein steady-state levels. Although our data show transcriptional control by Rb over Pak1, we could not yet rule out the possibility of Rb also acting on the half-life (*t*_1/2_) of Pak1 mRNA or protein. Therefore, we were also interested in determining whether Rb could affect these other aspects of gene expression. To test the effect of Rb on Pak1 mRNA *t*_1/2_, Rb+/+ and Rb-/- MC3T3 cells were cultured in the presence of the transcriptional inhibitor actinomycin D in order to inhibit further transcription. Cells were harvested for RNA extraction at 0, 5, 8, 10, 12, 17, 20, and 24 hours after actinomycin D addition. The extracted RNA was converted to cDNA and used in quantitative reverse transcription polymerase chain reaction (qRT-PCR) analyses to assay the diminution of Pak1 mRNA over the time course studied. Since production of new RNA was blocked by actinomycin D over the time course of this experiment, any changes in Pak1 mRNA levels resulted solely from degradation, and the rate of change allowed calculation of the *t*_1/2_ of Pak1 mRNA. As shown in Fig 3A, Pak1 mRNA was degraded with comparable decay constants (*k*) in both Rb+/+ and Rb-/- osteoblasts (0.061 ± 0.016 and 0.073 ± 0.014, respectively), indicating that Rb status does not significantly affect the kinetics of Pak1 mRNA degradation. Furthermore, the *t*_1/2_ of Pak1 mRNA in both Rb+/+ and Rb-/- osteoblasts was approximately 10 hours (11.3 and 9.5 hours, respectively), which is consistent with previous reports for Pak1 mRNA [21].

To ensure Pak1 mRNA degradation was not a reflection of general total RNA degradation, and thus poor sample quality, total RNA samples were obtained from various time points before converting them to cDNA and electrophoresed them in a 1.5% agarose gel containing formaldehyde to assess sample quality. As illustrated in Fig 3B, RNA samples showed robust, clearly discernible and defined 28S and 18S rRNA bands, and a 2:1 ratio of 28S-to-18S rRNA, indicating high quality of the RNA without signs of degradation. Therefore, the decay seen in Fig 3A is specific for Pak1 mRNA rather than a reflection of total RNA degradation. In addition, the kinetics of myeloid cell leukemia-1 (MCL1) mRNA decay was evaluated as a reference. MCL1 mRNA is known to be transient with a *t*_1/2_ of 2 hours [22]. Therefore, it was expected to show degradation during the time points chosen to evaluate Pak1 mRNA decay. MCL1 mRNA showed a *t*_1/2_ of 2.98 and 2.85 hours for Rb+/+ and Rb-/- MC3T3 cells, respectively (Fig 3C), approximating what has been reported previously [22]. MCL1 mRNA degraded with a *k* of 0.233 ± 0.051 for Rb+/+ and 0.243 ± 0.052 for Rb-/-. These results show that Rb status does not affect the *t*_1/2_ or the kinetics of MCL1 mRNA degradation.

Finally, we determined whether Rb could impinge upon Pak1 stability at the protein level. To test this, Rb+/+ and Rb-/- MC3T3 cells were incubated with 10 μg/mL puromycin in order to inhibit further translation and were then harvested at 0, 2, 5, 8, and 11 hours. In these experiments, however, drug toxicity precluded the analysis of time points beyond 11 hours. The resultant protein lysates were used for Western blot analysis to assay the diminution of Pak1 protein levels over the time course described above. Given that translation is blocked, any observed changes in Pak1 protein steady-state levels result solely from degradation, which in turn reflects changes in protein *t*_1/2_. α-Tubulin was used for normalization purposes to verify that the same quantity of protein was loaded in each lane. No significant changes were observed in terms of Pak1 protein degradation when comparing Rb+/+ and Rb-/- cells treated with puromycin (Fig 3D).

### The Pak1 Promoter Contains Rb-responsive Sites

**Fig 4:**
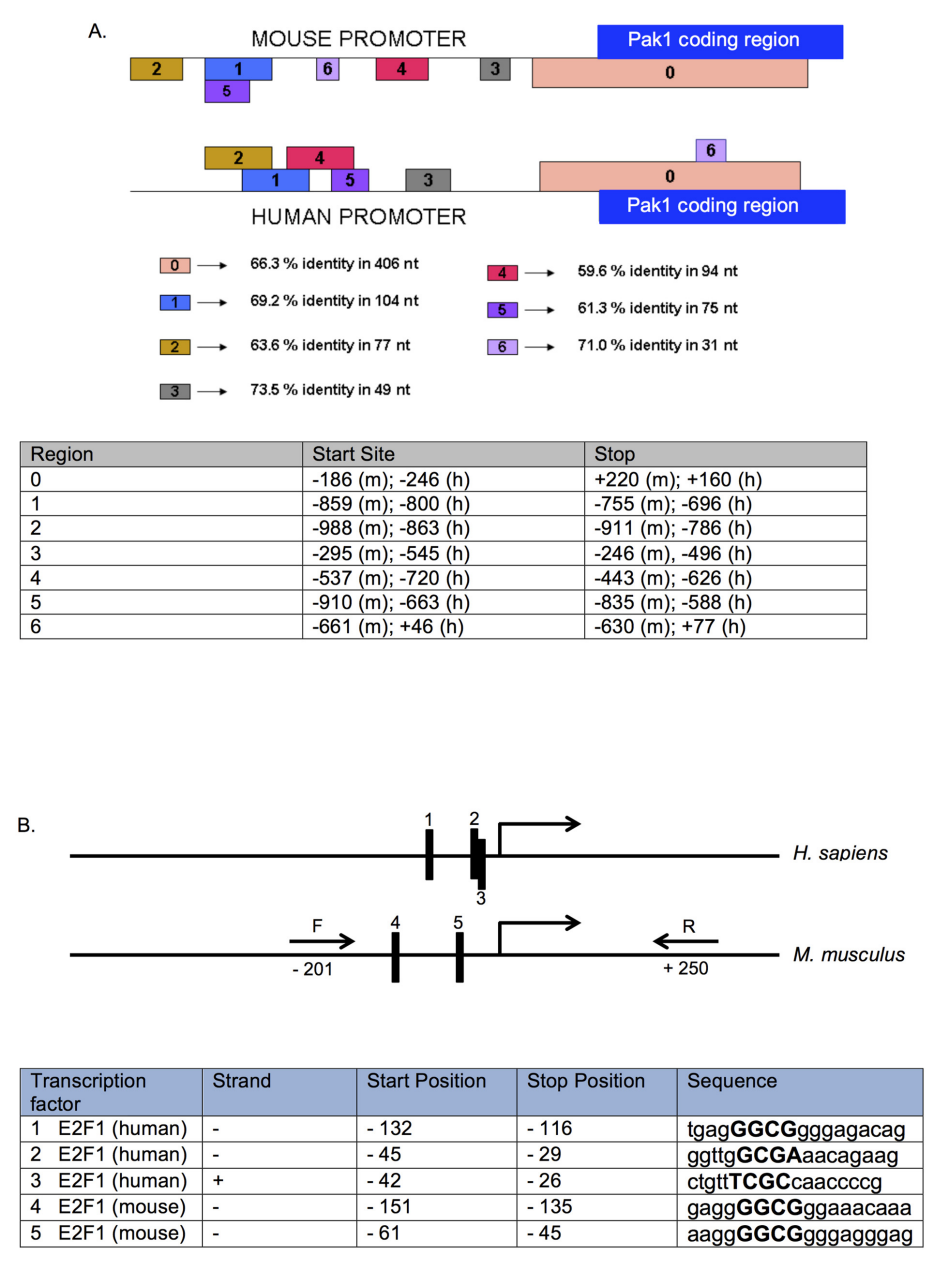
Conserved regions in the Pak1 promoter contain E2F binding sites. **(A)** Alignment of a region of 1,000 bp (from -700 to +300 bp) of the mouse and human Pak1 promoters generated by the Data Base of Transcriptional Start Sites (DBTSS), showing 7 conserved regions labeled 0–6. The longest conserved stretch is region 0, which spans a 406-bp region from -186 to +220 with 66% identity. The table below the promoter diagram shows the start and stop positions for each conserved region in mouse (m) and human (h). **(B)** Schematic of the human and mouse Pak1 promoters showing 5 E2F1 binding sites (labeled as 1–3 in the human promoter and 4 and 5 in the mouse promoter), as identified by Genomatrix analysis. These E2F1 binding sites are in the conserved region (labeled as 0 in **A)**. The table shows the positions, strand, and sequences of each E2F1 binding site, with the core nucleotides in each binding site indicated capitalized in bold. **(C)** A Pak1 mouse promoter-Firefly luciferase construct containing the 2 E2F binding sites was transfected into Rb+/+ and Rb-/- MC3T3 cells, and promoter activation was measured by its luciferase activity and normalized against a co-transfected Renilla luciferase construct. The Pak1 promoter region containing the E2F binding sites was amplified using the forward (F) and reverse (R) primers illustrated in **(B)** and in bold in S1 Fig. Transcriptional activity in Rb-/- cells was stronger than in Rb+/+ cells by a factor of 2.1 after normalization with Renilla luciferase, a value that is close to the 2.7-fold transcriptional induction that we observed in our nuclear run-on assays. *P < 0.05.

Nuclear run-on data (Fig 2) showed that the regulation of Pak1 levels by Rb is primarily transcriptional, with negligible contribution at the level of mRNA and protein stability (Fig 3). This is consistent with the well-characterized role of Rb as a transcriptional regulator. Given that transcriptional mechanisms account for the different Pak1 mRNA and protein expression levels between Rb+/+ and Rb-/- MC3T3 cells, we sought to characterize this transcriptional regulation in more depth, including the identification of the *cis-* and *trans-*elements involved in this regulation. We started the analysis by retrieving the human and mouse promoter sequences using the Cold Spring Harbor Laboratory Transcriptional Regulatory Element Database (TRED), defining as a putative promoter a 1,200-base pair (bp) region spanning from -1,000 to +200 bp relative to the transcriptional start site (S1 Fig). Next, two bioinformatics approaches were used to dissect the Pak1 promoter. First, the Data Base for Transcriptional Start Sites (DBTSS) aligned and compared the mouse and human promoters to identify highly conserved regions that are likely to contain *cis-*elements important for transcriptional regulation. DBTSS also identified transcriptional start sites in both promoters. Second, Genomatrix software identified transcription factor binding sites within the human and mouse Pak1 promoters, narrowing the search to transcription factors known for their capacity to interact with Rb. Combining these two approaches allowed us to better direct our search in the Pak1 promoter for Rb-responsive *cis-*regulatory elements as well as *trans*-co-regulators that could be important for the transcriptional repression of Pak1 by Rb. Fig 4A shows the sequence alignment of the human and mouse promoters produced by the DBTSS software. This analysis identified seven conserved regions between the human and mouse promoters, labeled 0–6. The conserved regions varied in length from as short as 31 bp (region 6) to as long as 406 bp (region 0), and their conservation ranged from 60% to 73% identity. The longest conserved stretch is region 0, which spans a 406-bp region from -186 to +220 with 66% identity. Region 0 not only shows sequence conservation between the mouse and human promoters, but it is also conserved in terms of its position within the *Pak1* gene structure of these two species, suggesting high regulatory importance.

To fulfill its function as a transcriptional regulator, Rb must interact with DNA-binding proteins that confer the Rb-containing complexes the capacity to bind to the promoters of target genes with high affinity and specificity. Therefore, a bioinformatics analysis was performed using Genomatrix to search the Pak1 promoter for binding sites for transcription factors known to form regulatory complexes with Rb. The Genomatrix analysis of the Pak1 promoter showed the highly conserved region 0 in Fig 4A to be rich in binding sites for Rb-interacting transcription factors. S1 Table shows the complete Genomatrix list of transcription factors that bind to the Pak1 promoter. One important novel finding of the Genomatrix analyses is that the E2F1 transcription factor, one of the best characterized binding partners of Rb, binds to both the human and mouse Pak1 promoters (S2 Fig and yellow highlights in S1 Table). Therefore, this study identifies Pak1 as a novel E2F target gene.

E2F1 is known predominantly as a transcriptional activator of proliferation-related genes. The anti-proliferative action of Rb requires repression of the trans-activating capacity of E2F [2]. This mechanism would explain the Pak1 transcriptional repression observed in Rb+/+ relative to Rb-/- cells. The Genomatrix analysis revealed three E2F1 binding sites in the human Pak1 promoter (Fig 4B, sites 1–3) and two E2F binding sites in the mouse promoter (sites 4 and 5 in Fig 4B and yellow highlights in S1 Fig), all of which are within 155 bp upstream of the transcriptional start site. We then PCR-amplified from mouse genomic DNA the region of the mouse Pak1 promoter containing the two E2F binding sites using the forward (F) and reverse (R) primers illustrated in Fig 4B and S1 Fig and cloned the resultant PCR product into a pGL3-MRP2 5’ UTR-luciferase vector and used this construct in luciferase reporter assays. Rb+/+ and Rb-/- MC3T3 cells were then co-transfected with the Pak1 promoter-Firefly luciferase reporter construct plus a Renilla luciferase reporter construct, and a dual luciferase assay was performed. As shown in Fig 4C, the cloned fragment of the Pak1 promoter containing the E2F binding sites retained its differential expression between Rb+/+ and Rb-/- cells. Transcriptional activity in Rb-/- cells was 2.1-fold stronger than in Rb+/+ cells after normalization with Renilla luciferase, a value approximating the 2.7-fold transcriptional induction observed in the nuclear run-on assays shown in Fig 2.

### Rb interacts with the Pak1 Promoter in Complex with E2F1

**Fig 5:**
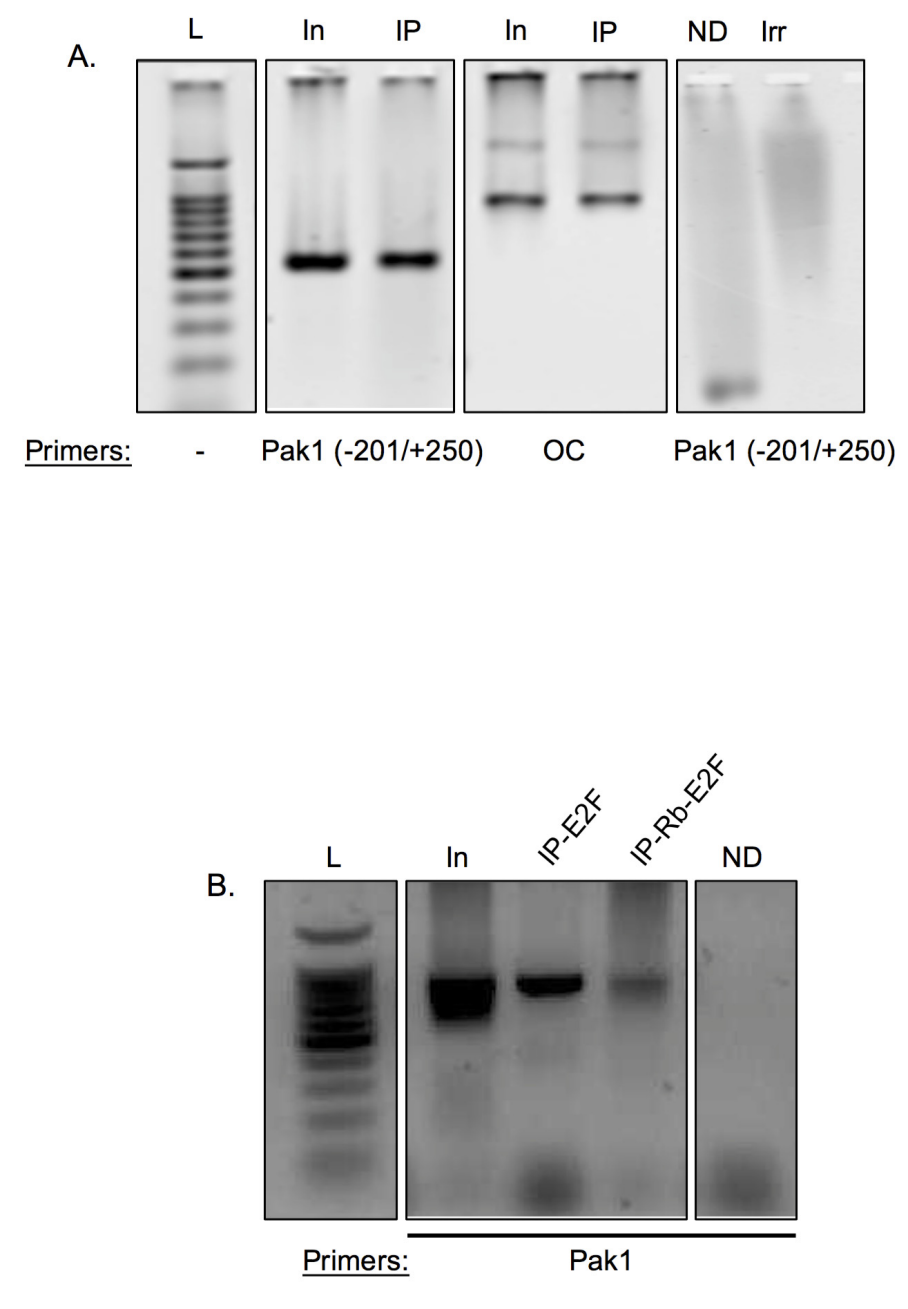
Rb interacts with the Pak1 promoter in complex with E2F. **(A)** ChIP assays showing interaction of Rb with the Pak1 promoter. The Pak1 promoter primer set used (-201/+250) is the same as illustrated in Fig 3B. The osteocalcin (OC) promoter was used as a positive control since Rb has been shown to physically interact with this promoter [4]. Negative controls were performed either by omitting DNA from the PCR reaction (no DNA or ND) or by immunoprecipitating with an irrelevant antibody against GAPDH. L, DNA ladder; In, PCR reactions done with input DNA not subjected to immunoprecipitation; IP, samples immunoprecipitated with anti-Rb antibody. **(B)** ChIP assays showing that Rb binds to the Pak1 promoter in complex with E2F1. ChIP assays in which the immunoprecipitation was done with anti-E2F1 antibody is labeled as IP-E2F. ChIP assays in which an immunoprecipitation done with an anti-Rb antibody followed by a second round of immunoprecipitation with an anti-E2F1 antibody is labeled as IP-Rb-E2F. PCR using primers against the Pak1 promoter resulted in a product, indicating interaction of the Rb-E2F complex to the Pak1 promoter. L, DNA ladder; In, PCR done with non-immunoprecipitated input DNA; ND, negative control with no DNA added to the PCR reaction.

The Pak1 promoter bioinformatics studies, nuclear run-ons, and luciferase transcriptional assays predict that, in Rb-expressing cells, a physical interaction exists between Rb, likely in complex with E2F, and the Pak1 promoter. To test this hypothesis, chromatin immunoprecipitation (ChIP) assays were performed in Rb+/+ MC3T3 osteoblasts. As shown in Fig 5A, immunoprecipitation of cross-linked extracts with an anti-Rb antibody followed by PCR using primers amplifying the Pak1 promoter (the -201/+250 primer set shown in Fig 4B) yielded a PCR product indicating an interaction between Rb and the Pak1 promoter. A fragment of the osteocalcin (OC) promoter was used as a positive control in these experiments given that Rb is known to interact with this promoter [4]. No PCR product was observed in negative controls, which were done by omitting the DNA from the PCR reaction (ND) or by immunoprecipitating the protein lysate with an irrelevant IgG.

Given the known weak and non-specific DNA binding capacity of Rb and identification of E2F1 binding sites in the Pak1 promoter, we predicted that the interaction between Rb and the Pak1 promoter is E2F1-mediated. To test this, we performed a ChIP in which the cross-linked DNA was immunoprecipitated with an anti-E2F1 antibody, followed by PCR using primers against the Pak1 promoter. This yielded a PCR fragment corresponding to the Pak1 promoter, confirming our prediction of the presence of E2F in such an interaction. (Fig 5B). Next, we performed a ChIP-on-ChIP experiment in which the immunoprecipitate obtained with the anti-Rb antibody was subsequently subjected to a second round of immunoprecipitation with the anti-E2F1 antibody. This second immunoprecipitation round was then used in a PCR reaction using primers against the Pak1 promoter. The PCR product obtained in this experiment (Fig 5B) demonstrates that E2F1, together with Rb, is part of a complex that binds to the Pak1 promoter.

### Pak1 Expression Is Upregulated in Several Tumor Types

**Fig 6:**
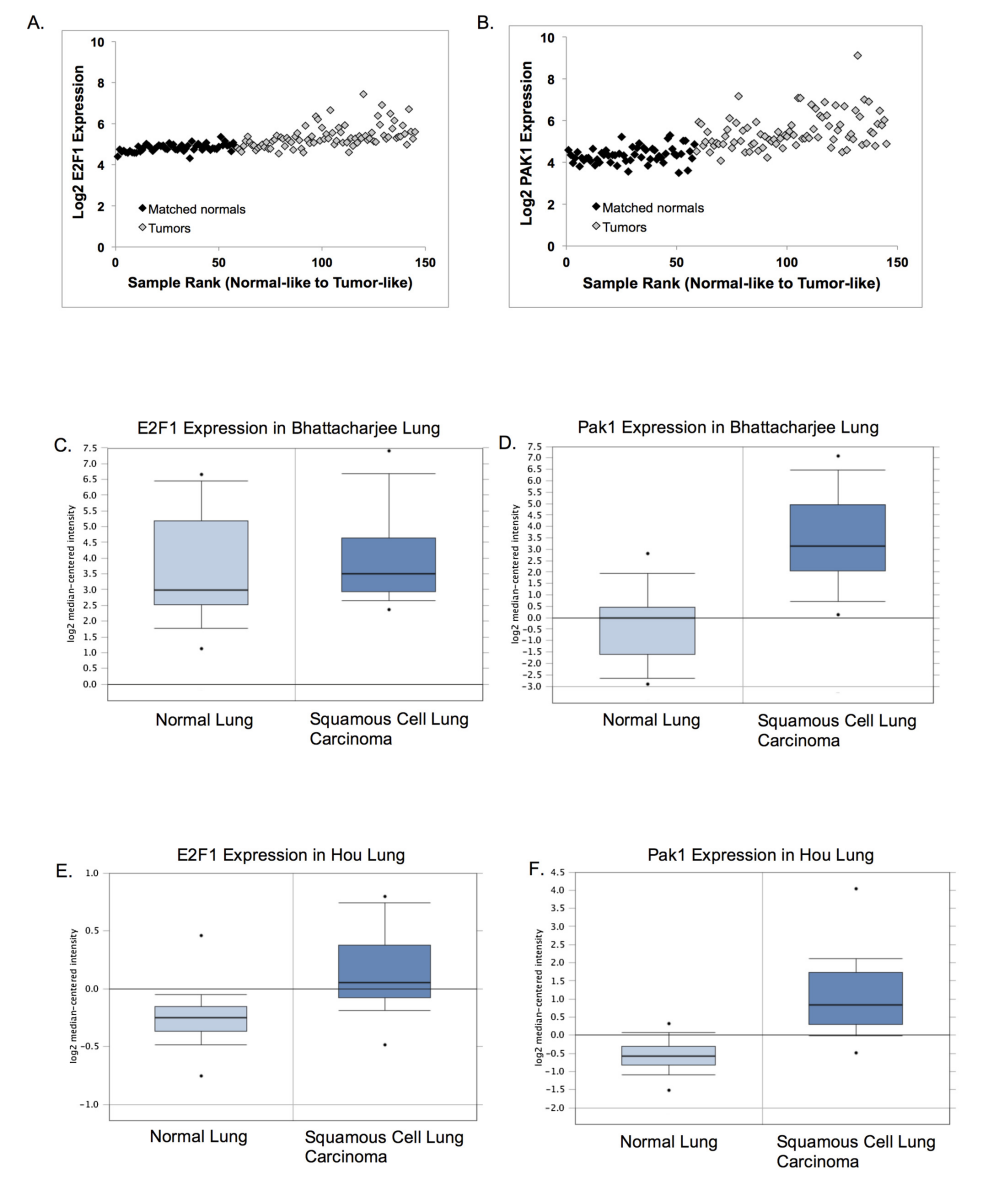
Changes in Pak1 expression occur in multiple solid tumors. **(A–J)** GSE19188 data sets for E2F1 **(A)** and Pak1 **(B)** expression in NSCLC tumor and adjacent normal samples were ranked from most normal to most tumor-like. Samples 1–58 are the adjacent normal tissues (black diamonds), whereas samples 59–145 represent tumor samples (light gray diamonds). Gene expression was determined via microarray for E2F1 **(C, G, E, I)** and Pak1 **(D, F, H, J)**. Oncomine (http://www.oncomine.org, Compendia Bioscience, Ann Arbor, MI, USA) was used for analysis and visualization of E2F1 and Pak1 expression in 4 common solid tumor types. **(C, D)** Analysis of E2F1 **(C)** and Pak1 **(D)** expression in normal lung versus squamous cell lung carcinoma from the Bhattacharjee lung database. **(E, F)** Analysis of E2F1 **(E)** and Pak1 **(F)** expression in normal lung versus squamous cell lung carcinoma from the Hou lung database. **(G, H)** Analysis of E2F1 **(G)** and Pak1 **(H)** expression in normal lung versus lung adenocarcinoma from the Stearman lung database. **(I, J)** Analysis of E2F1 **(I)** and Pak1 **(J)** expression in normal breast versus invasive breast carcinoma from the TCGA breast database.

We next analyzed E2F and Pak1 expression in public databases containing gene expression data for several solid tumor types. Due to the low incidence of osteosarcomas, which represents less than 1% of all the cancers diagnosed in the United States [23], no data on Pak1 expression on osteosarcomas were available. Instead, we examined four of the ten solid tumor types cataloged by the National Cancer Institute as the most common solid tumors. Analyses showed that Pak1 expression is increased in a statistically significant manner in all four tumor types, relative to matched normal tissues (Fig 6 and Table 1). One study [24] showed that Pak1 expression is increased 11.8-fold in squamous cell carcinoma tissue relative to matched normal tissue (Fig 6D). Other studies showed Pak1 up-regulation in cancer tissue over normal matched tissue of 2.96-fold in squamous cell lung carcinoma [25] (Fig 6F), 2.23-fold in lung adenocarcinoma [26] (Fig 6H), and of 2.16-fold in invasive breast carcinoma (TCGA invasive breast). Except for the Bhattacharjee study [24], which showed an 11.8-fold Pak1 increase in tumors versus normal tissue, the fold-inductions shown in the other studies are remarkably close to the 2.7-fold induction of Pak1 transcription upon Rb loss that we observed (Fig 2). In these studies, E2F1 expression was only slightly or not significantly induced in tumor tissue relative to matched normal tissue (Fig 6C, 6E and 6G), with the only exception being the invasive breast cancer study in which a 2.16-fold up-regulation of Pak1 correlated with a 2.7-fold up-regulation of E2F (Fig 6I).

**Table 1:** Oncomine was used for analysis of E2F1 and Pak1 expression in four common solid tumor types.

Database	E2F1 Fold Change	PAK1 Fold Change
Bhattacharjee lung	1.300 (0.231)	11.823 (2.24E-7)*
Hou lung	1.294 (8.84E-7)*	2.962 (1.47E-9)*
Stearman lung	1.227 (0.027)	2.227 (2.26E-8)*
TCGA invasive breast	2.734 (1.68E-22)*	2.156 (1.98E-16)*

The fold change value is determined by comparing the means of the two classes (tumor and normal adjacent tissue) in an analysis on a log2 scale and then converting that difference to a linear scale (http://www.oncomine.org, Compendia Bioscience, Ann Arbor, MI). P values are shown in parenthesis and significant changes in expression are denoted with an asterisk.

## Discussion

Regulation of cell adhesion is one of the latest roles ascribed to Rb, now widely regarded as a multifunctional protein. This is a relatively recently discovered role, considering that Rb has been predominantly studied for the past three decades as a cell cycle repressor. The role of Rb in cell adhesion is now gaining recognition as evidenced by the number of reports recently published on this topic. Early studies in the 1990s implicated Rb in cell adhesion by uncovering non-functional adherens junctions in osteosarcomas, retinoblastomas, and small-cell lung carcinomas, which are characterized by high frequencies of mutations in *RB1* [9, 10, 11]. Adherens junctions are cadherin- and catenin-containing membrane protein complexes that mediate cell-to-cell adhesion [27]. Studies demonstrate that their disruption, which is part of epithelial-to-mesenchymal transitions, exacerbates metastasis since it facilitates detachment of cancer cells from the primary tumor mass and the spread of tumor cells to near and distant tissues [20, 28]. Early studies linking Rb to cell adhesion were mostly correlative but were eventually expanded by the discovery of the underlying molecular mechanisms [16, 17, 29, 30, 31]. We have contributed to the understanding of Rb’s role in cell adhesion, specifically in osteoblasts, by showing that Rb expression is required for both cell-to-cell and cell-to-substrate adhesion [16, 17].

The published literature on Rb and cell adhesion points to the transcriptional induction of cadherin and integrin gene expression as one of the mechanisms by which Rb controls cell adhesion, in such a manner that Rb loss results in an aberrant expression profile of cell adhesion genes with consequent disruption of adherens junctions and alterations in cellular adhesion [16, 17, 29, 30, 31]. However, Rb appears to affect cell adhesion in mechanistically diverse manners that go beyond transcriptional regulation of cadherins and integrins. Early studies on Rb and cell adhesion showed that Rb-deficient tumors not only have diminished expression of adherens junction proteins but that these proteins are either mislocalized or that adherens junctions fail to anchor to the actin sub-membrane cytoskeleton [9, 10. 11], raising the possibility that Rb promotes the assembly of adherens junctions at the cell membrane as part of its tumor suppressive action. Consistent with this, our previous studies indicate that repression of the Rho GTPase Rac1 by Rb is required for the assembly and stabilization of adherens junctions at the cell membrane [16]. Unregulated Rac1 activity has been associated previously with oncogenic transformation, especially with cytoskeletal disarray, loss of cell polarity, and loss of cell adhesion [20, 28, 32], and thus we postulated that repression of Rac1 activity may be an additional tumor suppressive mechanism engendered by Rb. It is unlikely, however, that Rb controls Rac1 activity directly since Rb acts primarily by regulating gene transcription from its nuclear localization. Nevertheless, Rb could affect Rac1 activity indirectly by controlling the transcription of effectors or regulators of Rac1. Supporting this notion, we previously found that Rb expressing cells have dramatically reduced levels of the Rac1 effector, Pak1 [16]. However, our previous work did not address the mechanisms by which Rb affects Pak1 levels. In this article, we extended this observation by showing that, consistent with role of Rb as a transcriptional regulator, this repression occurs at the transcriptional level with minimal effects on the stability of Pak1 mRNA and protein stability. Our nuclear run-on and luciferase transcription assays showed a 2.7-fold increase in Pak1 transcription in Rb-/- cells relative to Rb+/+ cells, which is remarkably similar in magnitude to the Pak1 upregulation that we previously reported in Rb-/- cells using qRT-PCR and immunoblot approaches [16].

Regarding transcriptional regulation, Rb can be either a transcriptional activator or a repressor in a context-dependent manner. The role or Rb as a transcriptional activator has been predominantly documented in the context of differentiation, where Rb binds to, and promotes the trans-activating capacity of, the transcription factors that trigger tissue-specific gene expression. For example, Rb significantly upregulates MyoD transcriptional activity and induces expression of late muscle differentiation markers MHC and MCK [33]. Rb performs similar roles in adipogenic differentiation by activating C/EBP beta [34], and in osteoblast differentiation by activating Runx2 [4]. Therefore, the function of Rb is considered essential for the differentiation of these tissues. On the other hand, Rb represses transcription predominantly in the context of cell cycle regulation by binding to E2F transcription factors and abolishing their transactivating capacity [2, 3, 35]. This E2F/Rb complex represses transcription by blocking the transcriptional activating capacity of E2F and by recruiting histone deacetylases to silence the expression of genes required for DNA synthesis during S phase [36, 37]. Thus, the repressive effect of Rb on transcription is mediated predominantly by its interaction with E2F, and thus the presence of E2F binding sites in promoters is a hallmark of Rb-repressed genes. Consistent with this, and reinforcing the finding that Rb transcriptionally represses Pak1, we found E2F binding sites in both the human and mouse Pak1 promoters. Therefore, this study for the first time identifies Pak1 as a novel E2F target gene. Pak1 is a non-traditional E2F target in the sense that it is a non S-phase-related gene. However, this study is not the first to identify a non-traditional E2F target by such a definition. For example, matrix metalloprotease (MMP) genes such as *MMP9*, *MMP14*, and *MMP15*, which are usually overexpressed in non-small cell lung cancer, also have multiple E2F-binding sites in their promoters, and their expression is regulated by the Rb-E2F pathway [38]. Therefore, the universe of E2F target genes is expanding to include genes not related to S-phase progression. It is important to note that MMP overexpression has also been associated with metastasis [39, 40], and it may synergize with loss of cellular adhesion to exacerbate the invasive potential of cancer cells since MMPs are known to remodel the extracellular matrix and thus facilitate cell migration away from the primary tumor [38, 39, 40]. Therefore, although we have identified *Pak1* as a novel E2F target gene, there is a precedent for both non-cell cycle-related and metastasis associated E2F target genes. The E2F binding sites identified in the Pak1 promoter appear to be functional in terms of their capacity to confer Rb responsiveness to Pak1 transcription, as demonstrated by the luciferase transcriptional assays. Also, ChIP assays showed interaction of an Rb-E2F complex with such sites.

Consistent with the documented upregulated Rac1 activity in cancer [28], bioinformatics analyses showed that expression of the Rac1 effector, Pak1, is significantly upregulated in lung and breast solid tumors, relative to adjacent normal tissue, for at least a two-fold increase in all four of the databases that we analyzed. In the Bhattacharjee lung cancer database [24], Pak1 upregulation was observed to be nearly 12-fold, and in the Hou [25] and Stearman [26] lung cancer databases, Pak1 was upregulated 3- and 2.2-fold, respectively. Of note, Rb function is impaired in most, if not all, lung cancers, whether directly by mutations in the *RB1* gene itself or indirectly by mutations in the *CDKN2* locus coding for p16INK4a [41, 42], which result in Rb inactivation by hyperphosphorylation. Thus, Rb inactivation is a known driver of lung cancer. Although Pak1 upregulation in solid tumors was found in all four databases we studied, E2F was slightly upregulated only in the breast cancer database. However, this is not surprising given that mutations targeting E2F with possible effects on E2F expression are rarely documented in cancer [43].

As explained above, due to the low incidence of osteosarcomas, which represents less than 1% of all the cancers diagnosed in the United States [23], no data on Pak1 expression on osteosarcomas were found in public databases. This is in contrast to Pak1 expression data on solid tumors which is abundant. Nevertheless, there are several publications documenting altered expression of Pak proteins, their Rho GTPase regulatory partners, or their regulators in a variety of non-epithelial tumors. For example, Pak protein kinases are involved in Schwann cell transformation [44], and in a recent report, another member of the Pak family of kinases, Pak7, was found to be overexpressed in osteosarcomas [45]. This study further confirmed an oncogenic role for Pak1 expression in osteosarcoma. Pak7 silencing in the osteosarcoma cell line Saos-2 reduced proliferation and colony formation capacity, as well as the tumorigenic ability of xenografted Saos-2 [45]. Therefore, despite the fact that the low incidence of osteosarcomas has resulted in a paucity of Pak expression data in public databases, there are nevertheless several studies that support a role for Pak overexpression in the molecular etiology of non-epithelial tumors.

**Fig 7:**
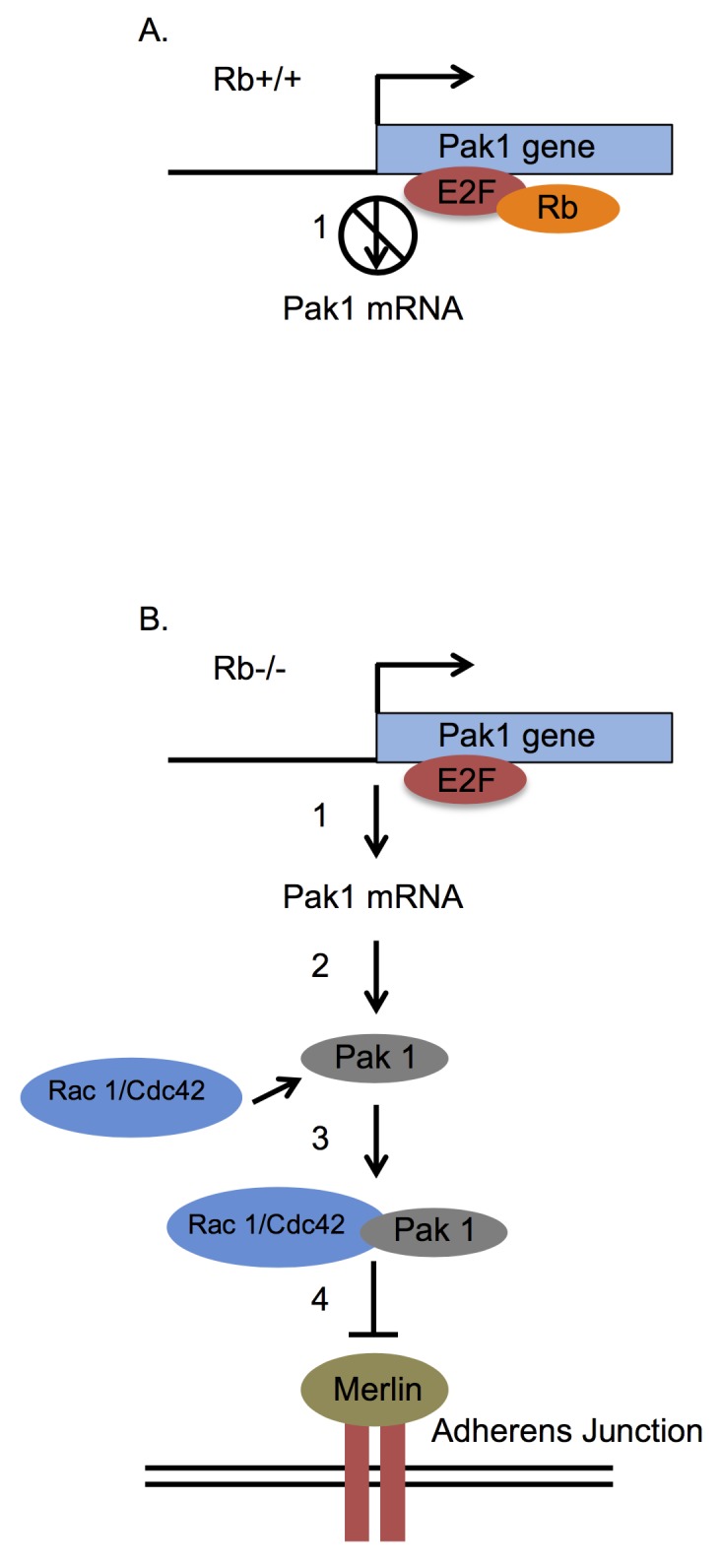
Model linking Rb function to cell adhesion via control of Pak1 expression. **(A)** Rb-expressing osteoblasts show significantly diminished Pak1 expression relative to their Rb-deficient counterparts since Rb binds to E2F1 in the Pak1 promoter and blocks its activity. This can be either by direct interference with E2F1’s trans-activating capacity or by recruitment of histone deacetylases to E2F1-sensitive promoters (*1*). **(B)** In the absence of Rb, the unrestricted E2F1 action induces transcription of the Pak1 gene (*1*) with consequent translation of the Pak1 protein (*2*). Once translated, Pak1 binds and is activated by a Rho GTPase such as Rac1 or Cdc42 to form an active complex (3), which then phosphorylates the merlin tumor suppressor in serine 518 (*4*), a phosphorylation that impairs merlin function. Because merlin acts mainly by promoting the stabilization of adherens junctions at the cell membrane, loss of merlin function by the Pak1-dependent phosphorylation will bring about a disruption of adherens junctions and therefore of intercellular adhesion.

The data presented in this article together with previous reports on Rb and osteoblast cell adhesion [16, 17] suggest the model illustrated in Fig 7. According to this model, in Rb-proficient cells, Rb binds to E2F and thus blocks E2F transactivating capacity with consequent abrogation of Pak1 expression (Fig 7A). In an Rb-null background, unrestricted E2F-mediated Pak1 transcription results in elevated levels of Pak1 transcripts and proteins (Fig 7B), as we previously documented [16]. Abundant Pak1 protein is then able to bind and become activated by its Rho GTPase partner and regulatory subunit, either Rac1 or Cdc42. Once in complex with Rac1, Pak1 phosphorylates the merlin tumor suppressor in serine 518, a phosphorylation that inactivates merlin and promotes its detachment from the cell membrane [16, 18, 19]. Given that merlin promotes assembly and stabilization of adherens junctions, merlin detachment from the cell membrane results in adherens junction disruption [16, 18, 19]. Of note, although Pak1 involvement in cell adhesion is not novel, and previous articles link Pak1 with cell adhesion-related pathways [46, 47], our work for the first time identifies Pak1 as being regulated by Rb in the control of cell adhesion.

The findings presented herein may have implications for osteoblast differentiation and osteosarcoma formation. Rb is required for osteoblast differentiation since it promotes osteoblast-specific gene expression by binding to and enabling the action of the osteoblast-specific transcription factor Runx2 [4, 48]. *RB1* inactivating mutations with consequent loss of Rb expression are observed in over 70% of sporadic osteosarcomas [49], and Rb loss has been shown to abrogate osteoblast differentiation [4, 48]. Interestingly, poor differentiation is a recurrent hallmark of osteosarcomas [50], suggesting that differentiation defects due to Rb loss could underlie the etiology of osteosarcomas. Rb loss could hamper osteoblast differentiation and promote osteosarcoma formation by a two-fold effect, first, by compromising the transactivating capacity of Runx2 with consequent attenuation of bone-specific gene expression, and second, by compromising cell-to-cell interactions, which are recognized as major drivers of osteoblast differentiation [13, 51, 52, 53, 54]. This is consistent with the previously published phenotype in a mouse model of osteosarcomas generated using a conditional knockout approach to selectively abrogate Rb expression in osteoblasts [16, 55].

Finally, a role for Rb in cell adhesion also has wider implications for carcinogenesis in general, specifically in explaining the highly aggressive behavior of some tumor types such as the ones characterized by Rb loss. The majority of osteosarcomas are poorly differentiated and already classified as high grade at the time of diagnosis, indicating their highly aggressive nature [50]. The poor prognosis of osteosarcoma patient is therefore unsurprising, with detectable metastases in 10%-20% of cases when diagnosed [50], and only approximately 10% of patients being able to achieve long disease-free time intervals [56]. The strong relation between Rb loss and osteosarcoma formation is demonstrated by the fact that inheriting mutant *RB1* alleles increases 1000-fold the risk of developing osteosarcoma, relative to the general population [57]. Also, mutations at the *RB1* locus are present in 60%-70% of both familial and sporadic osteosarcomas [49]. A dual role for Rb in both cell cycle control and cell adhesion could mechanistically explain how impairment of Rb function contributes to the aggressive nature of tumor types with high rates of Rb loss. In such tumors, complete Rb loss would result in a breakdown of cell cycle control, thus promoting initial tumor growth, as well as in a loss of cell-to-cell and cell-to-substrate contacts, which can exacerbate metastasis by promoting detachment of tumor cells from primary sites. This proclivity for early metastasis can be further enhanced if MMPs also overexpress due to Rb loss [38]. Conversely, tumors proficient in wild type Rb may still possess some residual Rb functionality that although not sufficient to override chronic proliferation, may be sufficient to preserve cellular adhesion, leading to the development of benign and spatially contained tumors. A role for Rb in cell adhesion mechanistically expands Rb’s arsenal of tumor suppressive tools, which would explain the potency of this preeminent tumor suppressor more adequately than the notion that Rb acts predominantly by repressing the cell cycle.

## Materials and Methods

### Cell lines

The cell lines used in these studies were Rb+/+ and Rb-/- MC3T3 osteoblasts. These cell lines were established and used during two previously published works [16, 17] and they have been previously described in details [16]. MC3T3 cells were maintained in alpha-Minimum Essential Medium supplemented with 10% fetal bovine serum and 1% penicillin-streptomycin and in a water-jacket incubator at 37^°^C with 5% CO_2_.

### Silencing of Pak1 expression and visualization of adherens junctions

MC3T3 cells were seeded in chambered culture slides and infected with a multiplicity of infection (MOI) of 1,000 with either Ad-CMV-RNAi scrambled control or Ad-m-Pak1-RNAi (Vector BioLabs). Following adenoviral infection, MC3T3 cells were further cultured for 14 days. Cells were then rinsed three times with 1x phosphate-buffered saline (PBS) and fixed in 4% formaldehyde diluted in 1x PBS for 15 min at room temperature. After fixation, chambers were washed three times in 1x PBS and were incubated with antibody buffer (1x PBS, 5% bovine serum albumin, and 0.3% Triton X-100) for 1 h at room temperature. Cells were then incubated overnight at 4°C with beta-catenin antibody (Cell Signaling, #8480) diluted 1:100 in antibody solution. Next day, chambers were washed three times with 1x PBS and then incubated for 1 h at room temperature with secondary anti-rabbit Alexa Fluor 555 (Cell Signaling, #4413) diluted 1:1000 in antibody solution. Finally, slides were rinsed three times with 1x PBS and mounted with ProLong Gold Antifade Mountant with DAPI (Life Technologies). Fluorescence was analyzed in a Nikon Eclipse 80i microscope equipped with a SPOT RT KE Camera.

### Nuclear Run-on Assays

Rb+/+ and Rb-/- MC3T3 osteoblasts were cultured until confluence and harvested for nuclei isolation using the Nuclei PURE Prep nuclei isolation kit by Sigma (# NUC-201), following manufacturer´s instructions. Briefly, 3.0 x 10^7^ cells in 10-cm culture plates were washed with PBS and lysed with ice-cold lysis solution consisting of 1 M dithiothreitol (DTT) and 10% Triton X-100. Nuclei were purified from lysates by centrifugation through a 1.8 M sucrose gradient for 45 minutes at 30,000 g at 4^°^C. After centrifugation, the supernatant containing cytoplasm and cell debris was carefully aspirated and the remaining nuclei pellet was re-suspended in Nuclei PURE storage buffer and kept at ^-^80°C until use. To generate the labeled probes, purified Rb+/+ and Rb-/- nuclei were subsequently used in *in vitro* transcription reactions to generate DIG-labeled mRNA using Roche’s DIG RNA labeling kit SP6/T7. DIG-11-UTP was used as the labeling nucleotide. Briefly, 5 X 10^7^ nuclei were mixed with an equal volume of 2X transcription buffer (10 mM Tris-HCL, pH 8.0, 5 mM MgCl_2_, 0.3 M KCl) and nucleotide solution (1 mM ATP, 1 mM CTP, 1 mM GTP, 0.6 mM UTP, 0.4 mM DIG-11-UTP, and 5 mM DTT), and *in vitro* transcription was allowed to proceed by incubating the reaction for 2 hours at 37°C with constant agitation. The reaction was terminated by adding 2 μL 0.2 M EDTA (pH 8.0). The DIG-labeled mRNAs to be used as probe were extracted with 3 volumes of Trizol and purified with Qiagen’s RNeasy kit according to manufacturer’s instructions. The purified DIG-labeled mRNAs were heat-denatured in Roche’s DIG Easy Hyb hybridization solution at 65^°^C for 10 minutes and stored in this same solution until use. The DNA dot blots were prepared by applying 1 μg of linearized, gel purified, alkali-denatured DNA into individual wells of a dot blot apparatus, and vacuum-transferring them to positively charged nylon membranes, followed by cross-linking using BioRad’s Strata-linker following the apparatus instructions for DNA cross-linking. One microgram of each of the following was immobilized to the nylon membranes: mouse Pak1 and GAPDH cDNAs, pSPT18, pSPT19 and HIV/Nef plasmids, and the unlabeled control sense RNA provided in the Roche’s DIG RNA labeling kit. Hybridization of DIG-labeled mRNAs to slot-blot nylon membranes was performed overnight in 5-mL screw-cap tubes at 50^°^C. After hybridization, membranes were washed twice in 2X saline sodium citrate (SSC)/0.1% sodium dodecyl sulfate (SDS) at room temperature for 10 minutes and then at 68^°^C for 20 minutes in 0.1X SSC/0.1% SDS. Chemiluminescense in the membranes was developed using Roche’s DIG luminescent detection kit, according to the manufacturer’s instructions, and detected and quantified by scanning membranes in BioRad’s ChemiDocTM XRS Molecular Imaging System.

### Pak1 mRNA stability assay

Rb+/+ and Rb-/- MC3T3 cells were incubated either with 5 μg/mL actinomycin D (Sigma) using DMSO as vehicle. Cells were harvested at 0, 5, 8, 10, 12, 17, 20, and 24 hours after actinomycin D addition by adding 1 mL of TRIZOL reagent per 10 cm^2^ of culture dish surface, and used for total RNA extraction using the ReliaPrep^®^ RNA cell mini prep system (Promega), following manufacturer’s instruction. As part of the RNA extraction procedure, samples were treated with DNase to eliminate genomic DNA contamination. One μg of RNA was then reverse transcribed using the iScript cDNA Synthesis Kit (Bio-Rad, Hercules, CA), according to manufacturer’s instructions. Pak1 and MCL1 mRNA expression levels were determined using TaqMan^®^ Assay (Applied Biosystems, Carlsbad, CA) as previously published [16]. Briefly, 50 ng of cDNA were subjected to 50 cycles of quantitative PCR in a RealPlex2 thermocycler (Eppendorf) using TaqMan^®^ Universal Mastermix according to the manufacturer’s instruction. Pak1 and MCL1 expressions were normalized to the β-actin reference gene, and relative expression levels of all genes were determined using the ^ΔΔ^Ct method. TaqMan^®^ assay primers used were Pak1 (Mm0044612_ml), ACTB (β-actin, Mm00607939_s1), and MCL1 (Mm01257351g1), using 15 pmol each. Data were analyzed by nonlinear regression, and the *t*_1/2_ was calculated from the first-order decay constant (*k*) obtained with the PRISM software program (GraphPad).

### Pak1 Protein Stability Assay

Rb+/+ and Rb-/- MC3T3 cells were cultured in the presence of 10 μg/mL puromycin (Sigma) in water, cells were harvested at 0, 2, 5, 8, and 11 hours after addition of the drug, and protein lysates were prepared as previously described [16]. Briefly, cells were lysed in 100–200 μL of ELB (50 mM HEPES, pH 7.2, 250 mM NaCl, 2 mM EDTA, 0.1% NP-40, 1 mM DTT) per 10-cm plate, plus protease and phosphatase inhibitors (1 mg/mL aprotinin, 1 μg/mL of leupeptin, 100 μg of phenylmethylsulfonyl fluoride, 4 mM sodium orthovanadate, 2 mM sodium pyrophosphate). Protein concentrations in cell lysates were determined by Bio-Rad protein assays. For immunoblotting, 50 μg of protein were separated by SDS-PAGE, transferred to nitrocellulose by standard procedures, and immunoblotted with anti-Pak1 (Santa Cruz, sc-882, rabbit polyclonal raised against the N-terminus of Pak1 of rat origin) and anti-α-tubulin (Calbiochem, CP06, mouse monoclonal raised against native chick brain microtubules) primary antibody at 1:100 dilutions. Proteins were detected by using horseradish peroxidase-conjugated donkey anti-mouse or donkey anti-rabbit secondary antibodies (Jackson ImmunoResearch Laboratories, Inc.) at 1:2,500 dilutions.

### Pak1 Promoter Bioinformatics

Human and mouse promoter sequences were retrieved from Cold Spring Harbor Laboratory TRED (http://rulai.cshl.edu/cgi-bin/TRED/tred.cgi?process=home), defining as a putative promoter a 1,200 bp region spanning from -1000 to +200 bp relative to the transcriptional start site. The TRED accession numbers for the human and mouse Pak1 promoters are 114750 and 76974, respectively. Sequence alignment and identification of transcriptional start sites were performed using DBTSS (http://dbtss.hgc.jp). Genomatrix (www.genomatix.de/en/index.html) was used to identify transcription factor binding sites within the human and mouse Pak1 promoters.

### Cloning of the Pak1 Promoter

A 502-bp region from mouse genomic DNA was PCR-amplified using the primers GGGCTCGAGTGCTCTAAAAAGCCTGTCTGG (forward) and GGGAAGCTTATGTTCTCCCCATTCCCTTC (reverse). This region corresponds to the mouse Pak1 promoter that contains the E2F binding sites. The PCR cycling conditions were 2 minutes of initial denaturation at 94°C followed by 33 cycles of 94°C for 30 seconds, 62°C for 30 seconds, and a final extension at 72°C for 2 minutes. Extensions containing two restriction enzyme sites, XhoI and HindIII, were included in each primer to facilitate cloning. The PCR products were purified with a PCR purification kit from QIAGEN (Valencia, CA), digested with XhoI and HindIII for 2 hours at 37°C, and ligated into the XhoI and HindIII-digested pGL3 MRP2 5′-UTR-luciferase plasmid (Promega) in order to generate the Pak1-luciferase reporter gene construct. Constructs were sequenced to verify their identities as Pak1 promoters.

### Dual Luciferase Reporter Assays

Rb+/+ and Rb-/- MC3T3 osteoblasts were cultured in 12-well plates without antibiotics. Liposome-mediated transfection was performed after 24 hours at 80–90% confluence. Briefly, 1.5 μg of the Pak1 promoter-Firefly luciferase reporter construct and 0.5 μg of Renilla luciferase reporter construct to control for transfection efficiency (Promega) were transfected into Rb+/+ and Rb-/- MC3T3 osteoblasts using Lipofectamine 2000 (Invitrogen) following manufacturer’s instructions. Cells were harvested 48 hours after transfection, and Firefly and Renilla luciferase activities were measured using the Dual Luciferase Assay System (Promega) according to manufacturer’s instructions. Bioluminescence was measured with a Turner Designs model TD-20/20 Luminometer. Bioluminiscence activity is represented as the ratio of Pak1-Firefly luciferase to Renilla luciferase activity.

### Chromatin Immunoprecipitation

Rb+/+ MC3T3 cells were cross-linked with 1% formaldehyde for 10 minutes at room temperature in a rocking platform, followed by quenching with 1.25 M glycine. Two hundred μL of nuclei preparation buffer and 100 μL of shearing buffer from the Nuclei Pure Prep nuclei isolation kit (Sigma) were added to cells in suspension, followed by vortexing and a 10-minute incubation in ice. Genomic DNA from lysed nuclei was sheared into fragments ranging from 1.0 to 1.5 kilobase-pairs (kbp), as assessed by 1% agarose gel, using a water bath sonicator for 10 minutes. Chromatin immunoprecipitation was done using the Imprint Chromatin IP kit (Sigma) following manufacturer´s instructions. Briefly, 1 μg of antibodies were immobilized into strip-wells provided by the kit in the presence of 100 μL of Antibody Buffer and mixing at 50–100 RPM on an orbital shaker for 60–90 min at room temperature. The antibodies used were anti-Rb, (BD Pharmingen 554136, mouse monoclonal, clone G3-245, against amino acids 332–344 of human Rb), anti-E21 (Santa Cruz sc-193, rabbit polyclonal against the C-terminus of human E2F1) and GAPDH (Calbiochem CB1001, mouse monoclonal, clone 6C5, raised against rabbit muscle GAPDH). The anti-GAPDH was used as the irrelevant IgG negative control. Sheared DNA was diluted 1:1 with the kit’s dilution buffer, and 100 μL of this dilution was added to each strip-well and incubated at room temperature for 60–90 minutes on an orbital shaker at 50–100 rpm. Cross-linking was reversed, and DNA was released by adding 40 μL of DNA release buffer containing proteinase K and 40 μL of reversing solution to each strip-well and incubating at 65°C for 90 minutes. The DNA purified from each well was subjected to PCR using primers to amplify a region of the Pak1 and Osteocalcin promoters. A 1% agarose gel was run to detect the presence of a PCR product.

### Mining of Solid Tumors Public Databases

Microarray studies conducted on cancers included in the National Cancer Institute’s list of the 10 most common solid tumors (bladder, breast, colon and rectal, endometrial, kidney (renal cell), lung, melanoma, pancreatic, prostate, thyroid; http://www.cancer.gov/cancertopics/types/commoncancers) were analyzed using Oncomine (http://www.oncomine.org, Compendia Bioscience, Ann Arbor, MI, USA). E2F1 and Pak1 probe-sets were analyzed, and data sets were ordered by under- or over-expression. P-values of E2F1 and Pak1 were analyzed. E2F and Pak1 expression levels in solid tumors were further analyzed using four previously described microarray studies to evaluate their mRNA expression [24, 25, 26].

## Supporting Information

S1 Fig Mouse promoter sequence retrieved from the Cold Spring Harbor Laboratory Transcriptional Regulatory Element Database (TRED). We defined as a putative promoter a 1,200-bp region spanning from -1,000 to +200 base pairs (bp) relative to the transcriptional start site (indicated in red). The forward and reverse primers we used to amplify the E2F binding site-containing fragment are indicated in bold and by (→) and (←), respectively. The two E2F binding sites are indicated by yellow highlight with the core nucleotides in bold.
(PDF)

S2 Fig The human and mouse Pak1 promoters contain E2F binding sites. Original schematic of the human and mouse Pak1 promoters produced by Genomatrix analysis. E2F binding sites, represented by green semicircles, can be seen in both promoters within 150 base pairs upstream of the transcriptional star sites.
(PDF)

S1 Table Genomatrix analysis reveals transcription factor binding sites in the Pak1 promoter. Original data produced by Genomatrix analysis showing all the transcription factors that bind to the Pak1 mouse and human promoters. E2F1 binding sites are highlighted in yellow.
(XLS)
