# Supplementary material for: The Retinoblastoma Tumor Suppressor Transcriptionally Represses Pak1 in Osteoblasts
Source: PLoS One. 2015 Nov 10;10(11):e0142406. doi: 10.1371/journal.pone.0142406 (PMC4640669; doi:10.1371/journal.pone.0142406)
Supplement: S1 Fig — We defined as a putative promoter a 1,200-bp region spanning from -1,000 to +200 base pairs (bp) relative to the transcriptional start site (indicated in red). The forward and reverse primers we used to amplify the E2F binding site-containing fragment are indicated in bold and by (→) and (←), respectively. The two E2F binding sites are indicated by yellow highlight with the core nucleotides in bold. (PDF) [file pone.0142406.s001.pdf]

-1,052	GCATAACTTG	ATTCCAGTCA	TGCGCCTCAA	GGGCAAGGGA	TGAGTTGGGT
-1,002	GAGTGTAGTG	AGTGATGATG	GGAGATACTG	ACTTCTGGGT	GTACAGGAAA
-952	TATGTGAAGG	TACATTTTTT	AACATTTCAG	TCTTAAGGAA	GCACCGTTTA
-902	TTGGGATGGG	GAAGACCAGA	GTTGTTTCTT	TAATGTGTCA	TCCAGCTCCA
-852	AGTTCTATTG	TAGTGACTGG	TATCTGACAA	GTGCTAAAGA	AATTCTGAGA
-802	GTGGTGTAGG	GCCTACTTCA	TAACTCTGTG	AAATAGACAT	AGGTTTGTAA
-752	AGTGTGGGGA	CACATAGTAA	ATAGCCCTTA	ACAAATAGTA	GCTGTTGCTG
-702	AACTTTCTCT	GTTCAAAGGA	GGTGGTTGGT	ATAGGGACCA	CCTTATTATC
-652	TTTCATGGGA	CACATTTTAG	AGAGTCAGCA	GAAAAGAATA	ATTTCCCCCT
-602	CTCCATTCTA	GTCTGAATGA	ATCTTGAATC	TAATTTGAGT	AGGTCTTGAT
-552	TCCGGGGTTC	AAGAGGACTC	CCACAGGCTC	TAGAACAGCT	GTTCTCAGCT
-502	TATAGGTCCA	GATCCCTTGA	GGGGTTGTTG	AACTACCCTT	TCACAGGGGT
-452	CACCTAAGAC	CATTGGAAAT	GTCAGATATT	TACATTACAA	TTCATAGCAG
-402	TAGCAAAATT	ACAGAATTAC	AAAATTATGA	AATAGCAACA	AAATTAATTT
-352	TATGATTGGG	GGTCACCACA	ATGTGAGGAA	CTATATTAAA	GAATCGCAGT
-302	GTTAGGAAGG	TTGAGAACTA	C**TGCTCTAAA**	**AAGCCTGTCT**	**GG**TACCTCTT
-252	GTTGGAGCCT	GGGTTGGGAG	AACAGCAGAG	ACAATCAGAA	CATTGGTTAT
-202	GTATTTGTTT	CC**CGCC**CCTC	AGCGCCTCTT	CAGAAAAAAA	GGGGCGGGGG
-152	CAGCGCTCTC	CCCGCCTCCT	TTCCTTCCTT	TCTCTGTTCC	TCCCTCCCTC
-102	**CCCGC**CCCTT	CACTAAGCCT	CTGTGT**CTTT**	**GCTGCCGCCC**	**TACGTGGGCC**
-52	**T**GAGTGTGCT	GCGCCTTGGG	AGCTGGGTGG	CTGCGCTGCG	GCGCTTAGCC
-2	GAATCCAGCC	TGTCAGGCTG	GTTCAGCCCG	GGCTTTGTCC	CAACTGCTGA
48	GCAGGGAGAG	GTGCGGCTCT	GCGACAGATA	CACAAGATCA	TCAGAGATGC
98	TGGGCCCCGG	ACCCACCGCC	AGGTAATGCT	GGCTTTCCCT	GGGCCTGGCA
148	TAGCTAGACT	GAGAGCTGGG	GTAGAAGGAG	CGGGTGTCTT	TCTGCCTTCC
198	GGG**GAAGGGA**	**ATGGGGAGAA**	**CATT**ACATTT	CACTTATATT	AAGGGATTC
