# Supplementary material for: The Retinoblastoma Tumor Suppressor Transcriptionally Represses Pak1 in Osteoblasts
Source: PLoS One. 2015 Nov 10;10(11):e0142406. doi: 10.1371/journal.pone.0142406 (PMC4640669; doi:10.1371/journal.pone.0142406)
Supplement: S2 Fig — Original schematic of the human and mouse Pak1 promoters produced by Genomatrix analysis. E2F binding sites, represented by green semicircles, can be seen in both promoters within 150 base pairs upstream of the transcriptional star sites. (PDF) [file pone.0142406.s002.pdf]

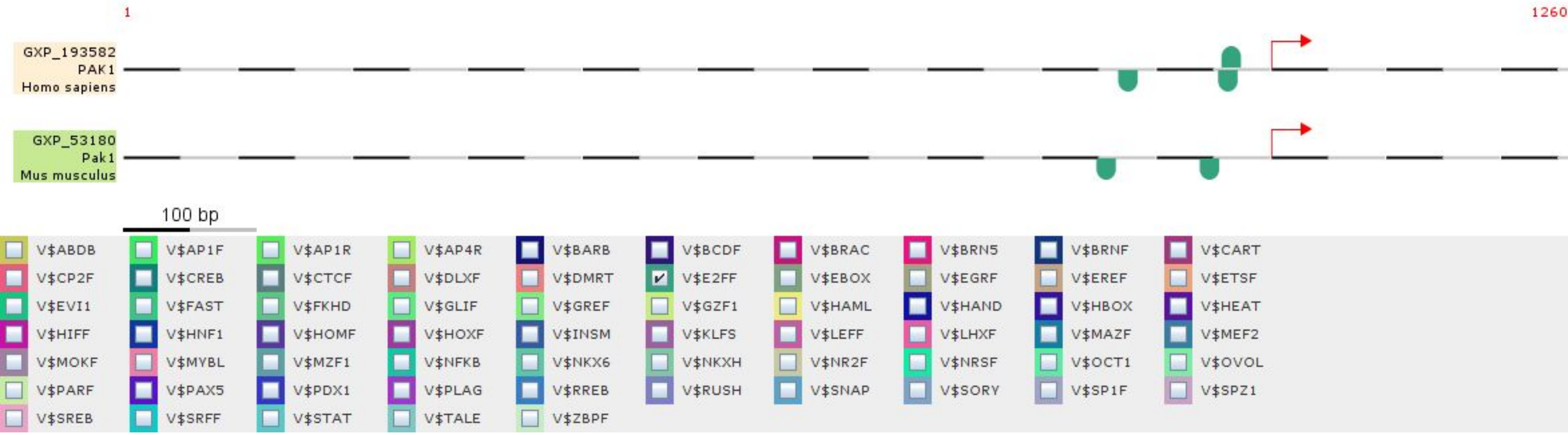
1
1260
GXP_193582
PAK1
Homo sapiens
GXP_53180
Pak1
Mus musculus
100 bp
V$ABDB
V$AP1F
V$AP1R
V$AP4R
V$BARB
V$BCDF
V$BRAC
V$BRN5
V$BRNF
V$CART
V$CP2F
V$CREB
V$CTCF
V$DLXF
V$DMRT
V$E2FF
V$EBOX
V$EGRF
V$EREF
V$ETSF
V$EVI1
V$FAST
V$FKHD
V$GLIF
V$GREF
V$GZF1
V$HAML
V$HAND
V$HBOX
V$HEAT
V$HIFF
V$HNF1
V$HOMF
V$HOXF
V$INSM
V$KLFS
V$LEFF
V$LHXF
V$MAZF
V$MEF2
V$MOKF
V$MYBL
V$MZF1
V$NFKB
V$NKX6
V$NKXH
V$NR2F
V$NRSF
V$OCT1
V$OVOL
V$PARF
V$PAX5
V$PDX1
V$PLAG
V$RREB
V$RUSH
V$SNAP
V$SORY
V$SP1F
V$SPZ1
V$SREB
V$SRFF
V$STAT
V$TALE
V$ZBPF
